# Supplementary material for: Genomic epidemiology offers high resolution estimates of serial intervals for COVID-19
Source: Nat Commun. 2023 Aug 10;14:4830. doi: 10.1038/s41467-023-40544-y (PMC10415581; doi:10.1038/s41467-023-40544-y)
Supplement: Supplementary file 1 — Supplementary Information [file 41467_2023_40544_MOESM1_ESM.pdf]

Supplemental Materials: Genomic epidemiology offers high resolution estimates of serial intervals for COVID-19

S1 Additional information, experiments and results

In this section we provide supplementary information and results, alongside several sensitivity experiments. Table S1 provides a translation between the wave 1 cluster labels used in this work, and those used in the original publication of the clusters in Seemann et al. [1]. Table S2 lists a selection of published wild-type COVID-19 serial interval estimates, for comparison against our cluster-specific estimates, which are provided in full in Table S3.

In Figure S1, we show the results of an experiment to compare use of a TN93 evolutionary model, as applied in this analysis, with a GTR+Γ4, as used to generate the wave 1 clusters in Seemann et al. [1]. Whilst TN93 and GTR+Γ4 are both reasonable viral evolutionary models, we chose to use TN93 in order to maintain a streamlined *R* codebase. In Figure S1, we see the difference in pairwise genomic distance between all clustered wave 1 case pairs is very similar under both evolutionary models, particularly for those closely related pairs we prioritize in this analysis. This is logical, given the low genomic diversity between our pairs.

In Table S4, we show the results of an experiment to explore the sensitivity of our results to the choice of transmission tree sampling approach. In the main text, we generated transmission networks by sampling an infector for each infectee according to the genomic distance and distance in symptom onset times between the pair. We compare this to a simpler model in which only genomic distance is considered. We find that the impact on our parameter estimates is minimal. Finally, in Figure S2 we explore the impact of the number of sampled transmission networks on our parameter estimates. We run the analysis using 50, 100, and 200 sampled transmission networks, and find minimal impact on the parameter estimates. This provides evidence that the

100 transmission networks used in the main text is sufficient.

Table S1: **Correspondence of wave 1 cluster names with cluster labels in Seemann et al. [1]**

Cluster	Seemann et al. [1] cluster
A1	4
A2	70
A3	74
A4	73
A5	7
A6	9
A7	19
A8	24
A9	76
A10	67

Table S2: **Published estimates of the COVID-19 serial interval, December 2019–June 2021, in days.**

Mean SI (95% CI)	Data, Country, Dates	Source
7.5 (5.3–19)	425 cases, Wuhan China, Dec 2019–Jan 2020	Li et al. 2020 [2]
4.0 (3.1–4.9)	28 pairs, World, Dec 2019–Feb 2020	Nishiura et al. 2020 [3]
5.8 (4.8–6.8)	77 pairs, China, Dec 2019–Mar 2020	He et al. 2020 [4]
6·3 (5·2–7·6)	48 pairs, Shenzhen China, Jan–Feb 2020	Bi et al. 2020 [5]
4.9 (3.6–6.2)	21 pairs, Hong Kong, Jan–Feb 2020	Zhao et al. 2020 [6]
4.17 (2.44–5.89)	93 cases, Singapore, Jan–Feb 2020	Tindale et al. [7]
4.31 (2.91–5.72)	135 cases, Tianjin China, Jan–Feb 2020	Tindale et al. [7]
5.54 (4.08–7.01)	28 pairs, Busan Korea, Jan–Mar 2020	Son et al. 2020 [8]
4.5 (3.1–5.5)	37 pairs, Iran, Feb–May 2020	Zare et al. 2021 [9]
4.0 (3.7–4.3)	471 pairs, Ireland, Apr-Dec 2020	McAloon et al. 2021 [10]
3.18 (2.55–3.81)	186 pairs (significant Alpha), UK, Sep 2020–Feb 2021	Geismar et al. 2021 [11]
2.3 (1.4–3.3)	68 pairs (Delta), Guangdong China, May–June 2021	Zhang et al. 2021 [12]

Table S3: **Full serial interval results table, for full set of 104 clusters.** Mean parameter estimates are presented with 95% confidence intervals in brackets.

**Wave 1 clusters**	**Serial interval mean μ**	**Serial interval SD σ**	**Sampling probability π**	**Proportion non-coprimary w**
A1	3.39 (1.43, 5.36)	1.93 (0.55, 3.31)	0.57 (0.38, 0.75)	0.55 (0.33, 0.77)
A2	5.95 (2.46, 9.45)	2.77 (0.61, 4.93)	0.60 (0.41, 0.78)	0.54 (0.34, 0.74)
A3	5.54 (2.08, 9.01)	2.55 (0.0, 5.92)	0.58 (0.38, 0.78)	0.56 (0.32, 0.80)
A4	4.01 (1.60, 6.42)	2.22 (0.69, 3.76)	0.62 (0.42, 0.82)	0.55 (0.32, 0.78)
A5	6.60 (2.48, 10.73)	2.39 (0.0, 4.81)	0.61 (0.41, 0.81)	0.53 (0.33, 0.74)
A6	4.85 (1.53, 8.16)	3.05 (0.0, 6.11)	0.56 (0.36, 0.76)	0.54 (0.33, 0.76)
A7	4.64 (2.44, 6.83)	2.61 (1.01, 4.21)	0.63 (0.46, 0.81)	0.59 (0.37, 0.81)
A8	3.40 (0.91, 5.90)	1.91 (0.35, 3.46)	0.58 (0.39, 0.77)	0.55 (0.32, 0.77)
A9	5.03 (2.07, 7.98)	2.69 (0.38, 4.99)	0.59 (0.40, 0.78)	0.56 (0.33, 0.78)
A10	3.42 (0.90, 5.94)	1.92 (0.29, 3.56)	0.58 (0.38, 0.78)	0.54 (0.32, 0.76)
**Total**	4.65 (1.06, 8.25)	2.38 (0.09, 4.67)	0.59 (0.40, 0.79)	0.55 (0.33, 0.78)
**Wave 2 clusters**	**Serial interval mean μ**	**Serial interval SD σ**	**Sampling probability π**	**Proportion non-coprimary w**
B1	4.85 (1.12, 8.58)	2.53 (0, 5.13)	0.57 (0.37, 0.77)	0.54 (0.31, 0.77)
B2	4.66 (1.52, 7.8)	2.49 (0.36, 4.62)	0.59 (0.4, 0.79)	0.55 (0.33, 0.78)
B3	7.67 (4.13, 11.21)	5.44 (1.94, 8.94)	0.58 (0.39, 0.77)	0.56 (0.34, 0.78)
B4	5.38 (1.1, 9.67)	2.28 (0.54, 4.02)	0.62 (0.41, 0.83)	0.52 (0.31, 0.74)
B5	8.31 (4.78, 11.85)	6.52 (3.12, 9.92)	0.6 (0.4, 0.79)	0.56 (0.33, 0.78)
B6	6.13 (2.09, 10.17)	3.52 (0.23, 6.81)	0.58 (0.38, 0.78)	0.55 (0.33, 0.77)
B7	5.8 (2.93, 8.66)	3.3 (1.42, 5.19)	0.62 (0.45, 0.8)	0.57 (0.34, 0.79)
B8	5.92 (1.97, 9.87)	3.55 (0.03, 7.08)	0.57 (0.37, 0.77)	0.55 (0.33, 0.78)
B9	7.29 (2.95, 11.63)	4.56 (0.36, 8.76)	0.57 (0.37, 0.76)	0.55 (0.33, 0.76)
B10	5.2 (3.06, 7.34)	3.05 (1.1, 5)	0.62 (0.45, 0.79)	0.6 (0.39, 0.82)
B11	5.87 (1.13, 10.6)	4.37 (0, 11.32)	0.52 (0.3, 0.73)	0.53 (0.31, 0.74)
B12	4.44 (0.02, 8.86)	2.02 (0.04, 4)	0.57 (0.37, 0.77)	0.52 (0.29, 0.75)
B13	3.85 (2.44, 5.25)	2.26 (1.14, 3.38)	0.63 (0.47, 0.79)	0.65 (0.45, 0.86)
B14	4.07 (0, 9.17)	1.64 (0.54, 2.73)	0.59 (0.4, 0.78)	0.52 (0.23, 0.82)
B15	4.78 (1.16, 8.39)	2.73 (0, 5.99)	0.56 (0.36, 0.76)	0.55 (0.32, 0.78)
B16	7.66 (4.86, 10.47)	4.82 (1.32, 8.32)	0.62 (0.45, 0.79)	0.58 (0.37, 0.79)
B17	5.37 (3.38, 7.36)	2.69 (0.82, 4.56)	0.64 (0.48, 0.8)	0.61 (0.42, 0.8)
B18	5.02 (2.13, 7.91)	3.02 (0.89, 5.15)	0.57 (0.38, 0.76)	0.54 (0.32, 0.76)
B19	6.7 (3.59, 9.8)	3.94 (1.95, 5.92)	0.67 (0.5, 0.83)	0.62 (0.39, 0.85)
B20	3.71 (0.68, 6.74)	2.02 (0.14, 3.9)	0.56 (0.36, 0.76)	0.54 (0.32, 0.75)
B21	3.88 (1.06, 6.7)	2.03 (0.03, 4.03)	0.56 (0.36, 0.76)	0.54 (0.32, 0.76)
B22	4.13 (1.31, 6.95)	2.3 (0.6, 4)	0.58 (0.39, 0.76)	0.55 (0.33, 0.77)
B23	6.84 (3.65, 10.03)	3.63 (0.06, 7.19)	0.59 (0.42, 0.77)	0.58 (0.35, 0.81)
B24	5.3 (2.36, 8.24)	2.98 (1.09, 4.87)	0.6 (0.42, 0.79)	0.56 (0.35, 0.78)
B25	3.45 (0.87, 6.04)	1.9 (0.17, 3.63)	0.58 (0.39, 0.78)	0.55 (0.32, 0.78)
B26	6.57 (2.46, 10.68)	4.26 (0, 8.85)	0.55 (0.35, 0.76)	0.55 (0.33, 0.76)

Wave 2 clusters	Serial interval mean μ	Serial interval SD σ	Sampling probability π	Proportion non-coprimary w
B27	6.44 (2.25, 10.64)	3.9 (0.53, 7.27)	0.58 (0.38, 0.78)	0.55 (0.32, 0.77)
B28	3.98 (0.65, 7.3)	2.32 (0.65, 3.99)	0.59 (0.39, 0.78)	0.54 (0.31, 0.77)
B29	3.58 (1.5, 5.65)	2.03 (0.59, 3.46)	0.61 (0.42, 0.8)	0.58 (0.35, 0.82)
B30	4.55 (2.14, 6.96)	2.58 (0.99, 4.18)	0.61 (0.42, 0.79)	0.58 (0.36, 0.8)
B31	5.7 (3.17, 8.22)	2.95 (0.97, 4.92)	0.6 (0.43, 0.77)	0.57 (0.37, 0.77)
B32	9.54 (0.82, 18.25)	6.11 (0, 12.9)	0.56 (0.35, 0.76)	0.53 (0.31, 0.76)
B33	6.08 (3.58, 8.58)	3.85 (1.88, 5.83)	0.63 (0.46, 0.8)	0.6 (0.39, 0.82)
B34	3.9 (0.66, 7.13)	1.98 (0.55, 3.42)	0.6 (0.41, 0.79)	0.55 (0.3, 0.8)
B35	8.82 (1.62, 16.02)	6.15 (0.13, 12.17)	0.56 (0.35, 0.76)	0.53 (0.32, 0.75)
B36	7.78 (3.63, 11.94)	4.66 (0.99, 8.32)	0.6 (0.4, 0.79)	0.57 (0.34, 0.79)
B37	5.63 (2.42, 8.85)	3.37 (0.02, 6.73)	0.54 (0.35, 0.73)	0.54 (0.33, 0.76)
B38	3.29 (0.49, 6.1)	1.73 (0.38, 3.08)	0.6 (0.4, 0.79)	0.54 (0.3, 0.79)
B39	4.38 (1.65, 7.11)	2.48 (0.7, 4.26)	0.54 (0.35, 0.73)	0.56 (0.33, 0.78)
B40	6.48 (2.5, 10.46)	3.84 (0.54, 7.14)	0.58 (0.39, 0.77)	0.55 (0.33, 0.78)
B41	4.66 (1.79, 7.52)	2.36 (0, 4.81)	0.59 (0.4, 0.78)	0.57 (0.33, 0.8)
B42	5.7 (2.6, 8.81)	3.23 (0.22, 6.24)	0.56 (0.36, 0.75)	0.56 (0.35, 0.78)
B43	2.64 (1.22, 4.06)	1.48 (0.6, 2.36)	0.6 (0.42, 0.78)	0.68 (0.44, 0.91)
B44	3.48 (0, 7.66)	1.61 (0.62, 2.61)	0.54 (0.34, 0.73)	0.51 (0.22, 0.8)
B45	4.68 (1.61, 7.74)	2.41 (0, 4.92)	0.55 (0.36, 0.74)	0.55 (0.33, 0.78)
B46	7 (3.66, 10.34)	3.26 (0.09, 6.44)	0.53 (0.35, 0.71)	0.54 (0.35, 0.74)
B47	6.74 (4.25, 9.22)	4.47 (1.79, 7.15)	0.61 (0.42, 0.79)	0.62 (0.41, 0.82)
B48	3.71 (1.57, 5.86)	2.11 (1.06, 3.17)	0.6 (0.43, 0.77)	0.62 (0.37, 0.87)
B49	4.23 (2.13, 6.34)	2.24 (0.55, 3.92)	0.59 (0.4, 0.77)	0.55 (0.35, 0.76)
B50	5.57 (2.6, 8.55)	3.25 (0.4, 6.09)	0.59 (0.4, 0.77)	0.57 (0.35, 0.79)
B51	5.46 (2.85, 8.07)	3.48 (0.76, 6.21)	0.55 (0.36, 0.74)	0.57 (0.35, 0.78)
B52	4.87 (1.5, 8.24)	2.28 (0.74, 3.82)	0.56 (0.37, 0.75)	0.51 (0.29, 0.74)
B53	3.86 (1.13, 6.6)	1.77 (0, 3.67)	0.47 (0.28, 0.65)	0.51 (0.29, 0.72)
B54	5.06 (2.62, 7.51)	2.87 (0.74, 5)	0.58 (0.39, 0.77)	0.57 (0.35, 0.79)
B55	3.33 (1.59, 5.07)	1.99 (0.81, 3.17)	0.57 (0.4, 0.75)	0.6 (0.36, 0.85)
B56	3.99 (0.24, 7.74)	1.71 (0.29, 3.13)	0.59 (0.41, 0.77)	0.55 (0.29, 0.82)
B57	6.74 (3.62, 9.86)	4.21 (0.93, 7.48)	0.58 (0.4, 0.77)	0.58 (0.36, 0.8)
B58	7.89 (4.18, 11.6)	4.2 (0.85, 7.55)	0.63 (0.45, 0.81)	0.57 (0.35, 0.79)
B59	5.24 (1.34, 9.13)	2.85 (0, 6.01)	0.55 (0.35, 0.76)	0.53 (0.32, 0.75)
B60	2.75 (0.53, 4.97)	1.51 (0.2, 2.82)	0.59 (0.4, 0.79)	0.55 (0.31, 0.79)
B61	4.35 (0, 9.66)	1.71 (0.45, 2.97)	0.58 (0.38, 0.77)	0.51 (0.25, 0.76)
B62	3.96 (0, 8.26)	1.78 (0.36, 3.2)	0.59 (0.39, 0.78)	0.53 (0.28, 0.78)
B63	3.39 (0.4, 6.38)	1.93 (0, 3.92)	0.53 (0.33, 0.73)	0.53 (0.31, 0.74)
B64	3.78 (1.31, 6.25)	2 (0.29, 3.71)	0.57 (0.39, 0.76)	0.56 (0.33, 0.8)
B65	4.61 (1.56, 7.66)	2.49 (0.62, 4.36)	0.57 (0.38, 0.76)	0.56 (0.33, 0.78)
B66	4.66 (0.93, 8.38)	2.37 (0, 4.82)	0.58 (0.38, 0.77)	0.54 (0.31, 0.77)
B67	5.26 (2.96, 7.57)	2.89 (1, 4.78)	0.62 (0.44, 0.79)	0.59 (0.36, 0.81)
B68	2.8 (0.34, 5.27)	1.4 (0.14, 2.67)	0.59 (0.39, 0.79)	0.54 (0.31, 0.77)

**Wave 2 clusters**	**Serial interval mean** μ	**Serial interval SD** σ	**Sampling probability** π	**Proportion non-coprimary** w
B69	5.36 (2.7, 8.02)	2.88 (0.54, 5.22)	0.58 (0.4, 0.76)	0.57 (0.35, 0.78)
B70	4.56 (2.25, 6.87)	2.56 (0.85, 4.26)	0.63 (0.45, 0.81)	0.6 (0.37, 0.84)
B71	4.04 (0.8, 7.27)	1.9 (0.55, 3.25)	0.46 (0.26, 0.65)	0.49 (0.27, 0.71)
B72	4.39 (1.29, 7.5)	2.31 (0.31, 4.32)	0.53 (0.33, 0.72)	0.54 (0.32, 0.76)
B73	1.97 (0, 3.95)	0.92 (0.03, 1.8)	0.59 (0.4, 0.78)	0.56 (0.31, 0.81)
B74	9.33 (3.17, 15.48)	6.2 (0.59, 11.82)	0.57 (0.37, 0.78)	0.55 (0.32, 0.77)
B75	3.76 (1.56, 5.96)	2.01 (0.51, 3.51)	0.57 (0.38, 0.76)	0.56 (0.33, 0.78)
B76	3.73 (0, 7.81)	1.69 (0.22, 3.17)	0.55 (0.36, 0.75)	0.53 (0.29, 0.76)
B77	7 (2.16, 11.84)	5.33 (0, 11.21)	0.54 (0.33, 0.75)	0.54 (0.32, 0.75)
B78	5.8 (2.29, 9.31)	2.86 (0.46, 5.26)	0.56 (0.37, 0.75)	0.53 (0.32, 0.74)
B79	4.12 (1.32, 6.91)	2.25 (0.49, 4.01)	0.58 (0.38, 0.78)	0.54 (0.32, 0.76)
B80	6.91 (3.15, 10.66)	3.78 (0.43, 7.13)	0.58 (0.39, 0.77)	0.55 (0.34, 0.77)
B81	6.66 (1.94, 11.37)	4.72 (0, 10.71)	0.54 (0.33, 0.74)	0.54 (0.32, 0.76)
B82	2.22 (1.26, 3.19)	1.32 (0.42, 2.23)	0.5 (0.33, 0.67)	0.56 (0.32, 0.8)
B83	6.79 (3.23, 10.35)	4.44 (1.14, 7.73)	0.59 (0.39, 0.78)	0.55 (0.33, 0.76)
B84	4.42 (1.22, 7.62)	2.41 (0, 4.85)	0.58 (0.38, 0.78)	0.54 (0.32, 0.77)
B85	5.91 (2.49, 9.34)	2.86 (0.24, 5.49)	0.6 (0.41, 0.8)	0.56 (0.33, 0.78)
B86	4.31 (0, 9.63)	1.46 (0.35, 2.56)	0.57 (0.38, 0.77)	0.49 (0.23, 0.75)
B87	4.99 (1.85, 8.12)	2.78 (0.12, 5.44)	0.58 (0.38, 0.77)	0.55 (0.33, 0.78)
B88	3.82 (0.23, 7.41)	1.9 (0.3, 3.49)	0.58 (0.38, 0.77)	0.54 (0.3, 0.78)
B89	4.4 (2.44, 6.37)	2.46 (0.68, 4.24)	0.6 (0.42, 0.77)	0.6 (0.38, 0.83)
B90	3.74 (1.79, 5.69)	2.11 (0.66, 3.57)	0.62 (0.44, 0.8)	0.6 (0.37, 0.83)
B91	5.75 (0.23, 11.28)	1.95 (0.43, 3.48)	0.59 (0.39, 0.8)	0.48 (0.24, 0.72)
B92	4.18 (1.5, 6.86)	2.22 (0.13, 4.3)	0.6 (0.4, 0.79)	0.56 (0.33, 0.78)
B93	3.94 (1.68, 6.2)	2.12 (0.38, 3.86)	0.6 (0.41, 0.78)	0.57 (0.34, 0.8)
B94	2.96 (0.17, 5.74)	1.5 (0.23, 2.78)	0.6 (0.41, 0.79)	0.55 (0.31, 0.8)
**Total**	5.17 (0.47, 9.87)	2.95 (0, 6.67)	0.58 (0.38, 0.78)	0.56 (0.32, 0.79)

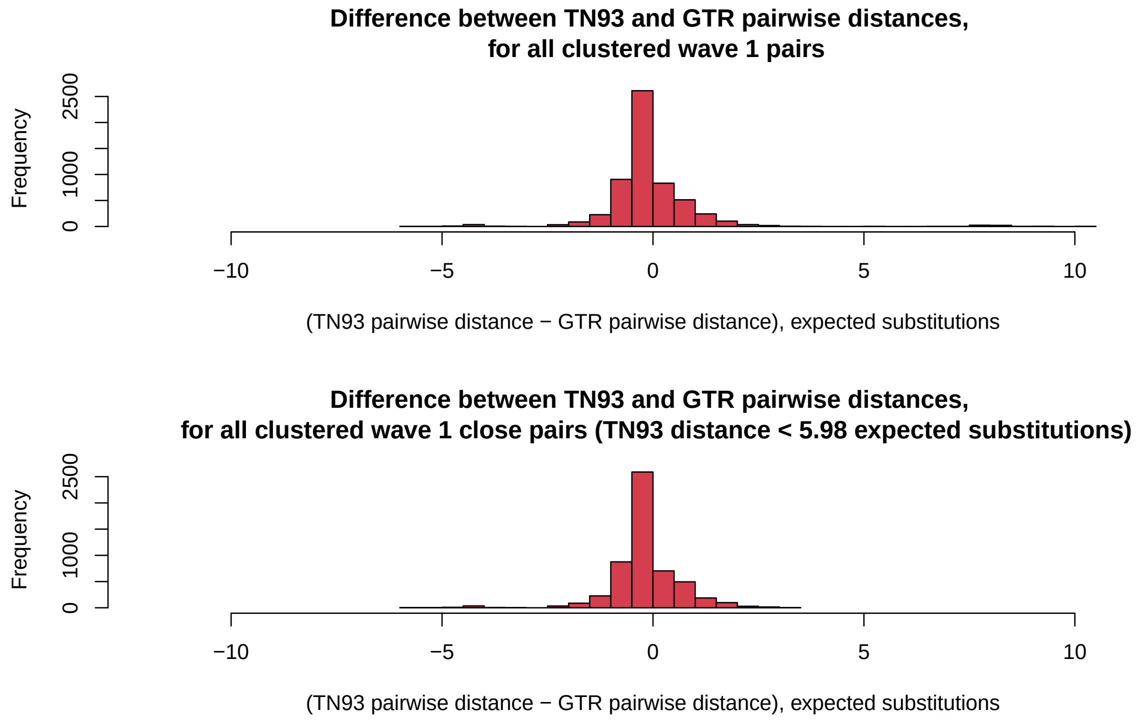

Figure S1: **Comparison of pairwise genetic distances under TN93 and GTR+Γ4 models, among wave 1 within-cluster pairs.** Distances in units of expected substitutions per sequence. ‘Close’ pairs defined as a TN93 distance less than 5.98 expected substitutions. For most pairs, particularly those close pairs we focus on in this analysis, the TN93 and GTR+Γ4 distances are very similar, with 0–1 expected substitutions difference.

Table S4: **Sensitivity analysis to the choice of transmission tree sampling approach (picking an infector for each infectee).** Method 1: approach from main text, in which sampling probability is inversely proportional to onset date difference and genomic distance. Method 2: sampling probability is calculated from genomic distance only. Results shown are mean parameter estimates and 95% confidence intervals.

**Method 1**	**Serial interval mean** μ	**Serial interval SD** σ	**Sampling probability** π	**Proportion non-coprimary** w
A1	3.39 (1.43, 5.36)	1.93 (0.55, 3.31)	0.57 (0.38, 0.75)	0.55 (0.33, 0.77)
A2	5.95 (2.46, 9.45)	2.77 (0.61, 4.93)	0.60 (0.41, 0.78)	0.54 (0.34, 0.74)
A3	5.54 (2.08, 9.01)	2.55 (0.0, 5.92)	0.58 (0.38, 0.78)	0.56 (0.32, 0.80)
A4	4.01 (1.60, 6.42)	2.22 (0.69, 3.76)	0.62 (0.42, 0.82)	0.55 (0.32, 0.78)
A5	6.6 (2.48, 10.73)	2.39 (0.0, 4.81)	0.61 (0.41, 0.81)	0.53 (0.33, 0.74)
A6	4.85 (1.53, 8.16)	3.05 (0.0, 6.11)	0.56 (0.36, 0.76)	0.54 (0.33, 0.76)
A7	4.64 (2.44, 6.83)	2.61 (1.01, 4.21)	0.63 (0.46, 0.81)	0.59 (0.37, 0.81)
A8	3.40 (0.91, 5.90)	1.91 (0.35, 3.46)	0.58 (0.39, 0.77)	0.55 (0.32, 0.77)
A9	5.03 (2.07, 7.98)	2.69 (0.38, 4.99)	0.59 (0.40, 0.78)	0.56 (0.33, 0.78)
A10	3.42 (0.90, 5.94)	1.92 (0.29, 3.56)	0.58 (0.38, 0.78)	0.54 (0.32, 0.76)
Total	4.65 (1.06, 8.25)	2.38 (0.09, 4.67)	0.59 (0.40, 0.79)	0.55 (0.33, 0.78)
**Method 2**	**Serial interval mean** μ	**Serial interval SD** σ	**Sampling probability** π	**Proportion non-coprimary** w
A1	3.39 (1.5, 5.28)	1.94 (0.56, 3.31)	0.58 (0.39, 0.77)	0.56 (0.33, 0.78)
A2	7.37 (4.88, 9.86)	2.56 (0.35, 4.78)	0.62 (0.47, 0.76)	0.52 (0.36, 0.68)
A3	5.52 (2.15, 8.88)	2.53 (0, 6.03)	0.59 (0.39, 0.79)	0.57 (0.32, 0.82)
A4	4.11 (1.77, 6.45)	2.18 (0.66, 3.71)	0.63 (0.43, 0.82)	0.56 (0.33, 0.79)
A5	6.79 (2.33, 11.24)	2.77 (0, 5.66)	0.6 (0.4, 0.8)	0.55 (0.33, 0.76)
A6	4.41 (1.68, 7.15)	2.5 (0, 5.37)	0.55 (0.35, 0.74)	0.55 (0.33, 0.76)
A7	4.66 (2.78, 6.53)	2.54 (0.89, 4.19)	0.63 (0.46, 0.8)	0.59 (0.38, 0.8)
A8	2.98 (1.09, 4.86)	1.84 (0.53, 3.14)	0.56 (0.37, 0.75)	0.54 (0.32, 0.76)
A9	5.31 (2.12, 8.5)	2.97 (0.48, 5.46)	0.59 (0.4, 0.78)	0.56 (0.33, 0.78)
A10	4.11 (0.85, 7.36)	1.85 (0.11, 3.59)	0.6 (0.41, 0.8)	0.55 (0.32, 0.77)
Total	4.63 (1.08, 8.18)	2.36 (0, 4.75)	0.59 (0.4, 0.79)	0.56 (0.33, 0.78)

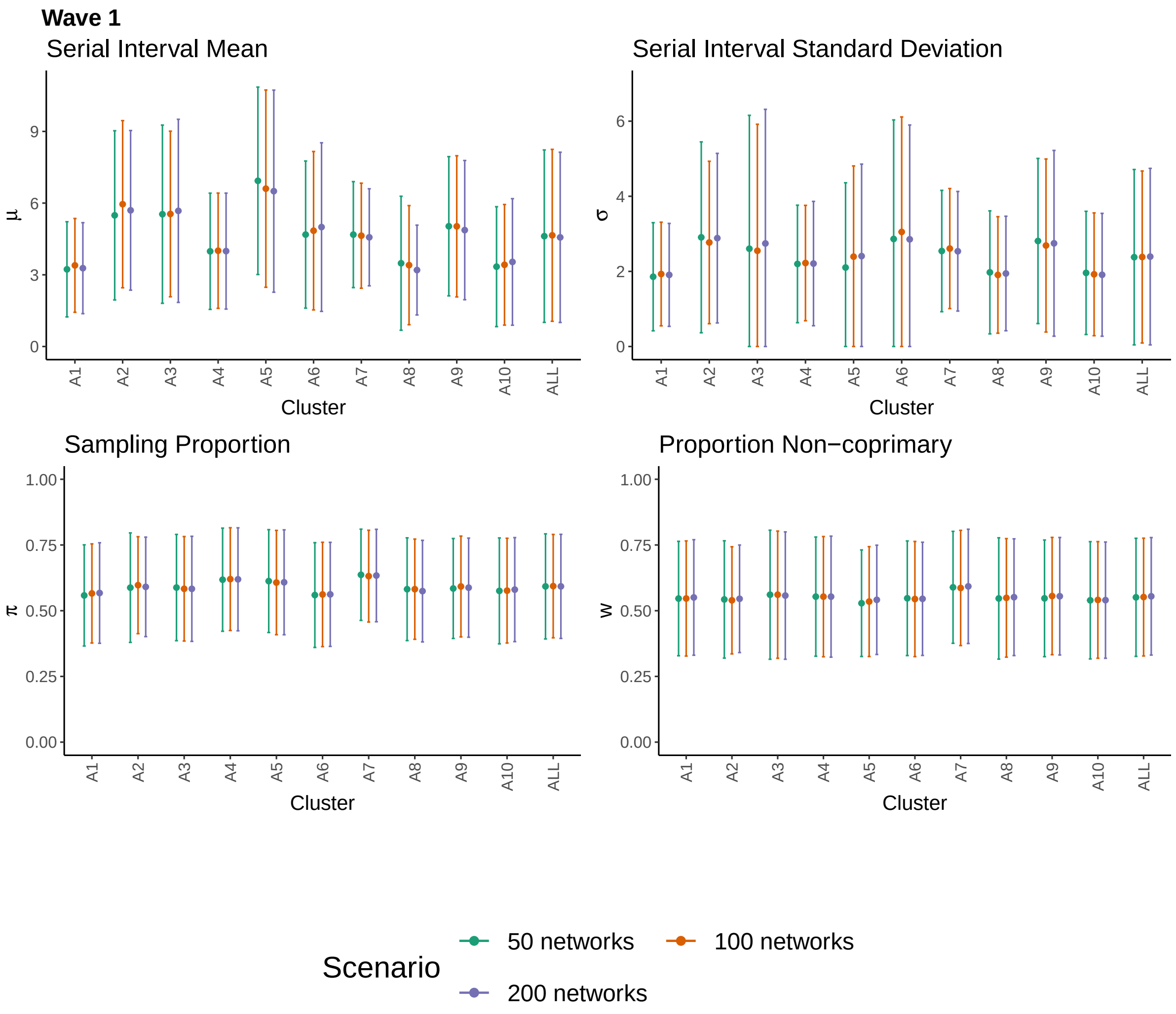

Figure S2: **Results of sensitivity analysis for the number of sampled transmission networks.** Estimates of the model parameters (μ, σ, π, w) for the wave 1 clusters (with size as defined in Table 1) are shown, when 50, 100, and 200 transmission networks are sampled. Mean estimates are shown as points, and 95% confidence intervals as bars. Sampling 50–200 networks does not meaningfully change the parameter estimates.

S2 Sensitivity to prior distribution

In this section, we explore the model sensitivity to the assumed prior distributions for sampling probability π and proportion non-coprimary w. In the main text, we use a Beta$(12, 11)$ prior distribution for both parameters. Here, we repeat the analysis of wave 1 clusters under 4 different prior distribution scenarios for each of π and w: increased/decreased mean and increased/decreased standard deviation. These prior distribution scenarios are plotted in Figure S3. For each scenario we sample 100 transmission networks in each of the 10 wave 1 clusters, as before.

Results of the sensitivity analysis are shown in Figure S4 and Figure S5. We find that the estimates of serial interval mean μ and standard deviation σ are relatively robust to changes in the sampling priors (that is, the prior distributions for parameters π and w). The relative ordering of clusters is largely preserved. The estimates of π and w are changed substantially in line with their prior. These results suggest that the data are sufficiently informative of the underlying serial interval distribution, so long as we have some minimal understanding of the case ascertainment rate.

We additionally run a simulation study to explore the impact of choosing an incorrect, or a more/less variable, prior on π and w. We use the same simulation model as in the main text (see Methods for full details), simulating outbreaks in a population of 200 individuals with a $\Gamma(\mu = 4.5, \sigma = 2)$ serial interval distribution. We down-sample 50% of cases before running the serial interval estimation, in which we sample 100 transmission networks per outbreak, for a comparable setting and cluster size to the COVID-19 analysis. We explore how our estimates of the model parameters vary under 7 different prior distributions on π and w, using the same logic as in the main text that both parameters are related to the sampling proportion $p = 0.5$. Our baseline prior is therefore Beta distributed with mean 0.5 and standard deviation 0.1. The 7 prior scenarios are described in Table S5 and plotted in Figure S6

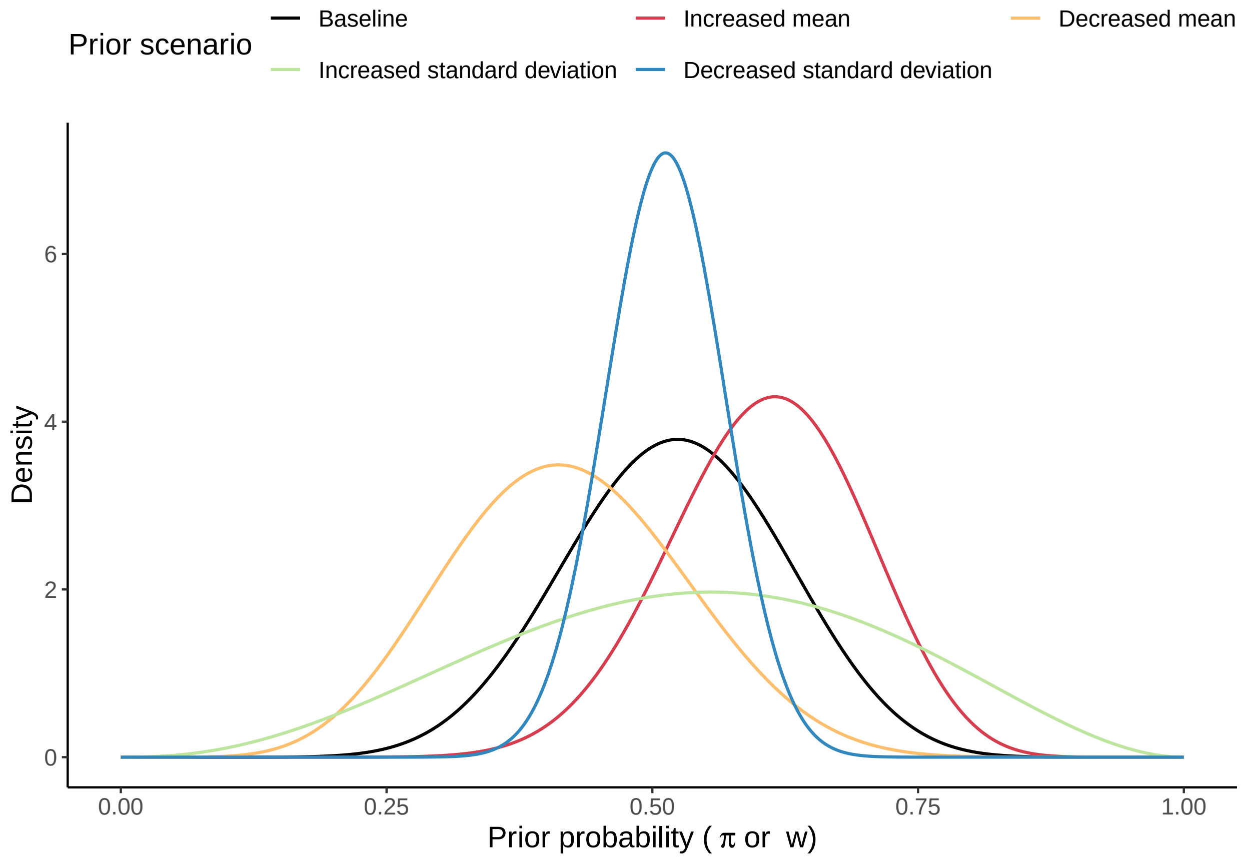

Figure S3: **Four scenarios for exploring sensitivity to the prior distribution of π and w, compared to a baseline Beta(12,11) prior from main analysis.** Increased mean: Beta(17,11), decreased mean: Beta(8,11), increased standard deviation: Beta(3.5,3), decreased standard deviation: Beta(42,40).

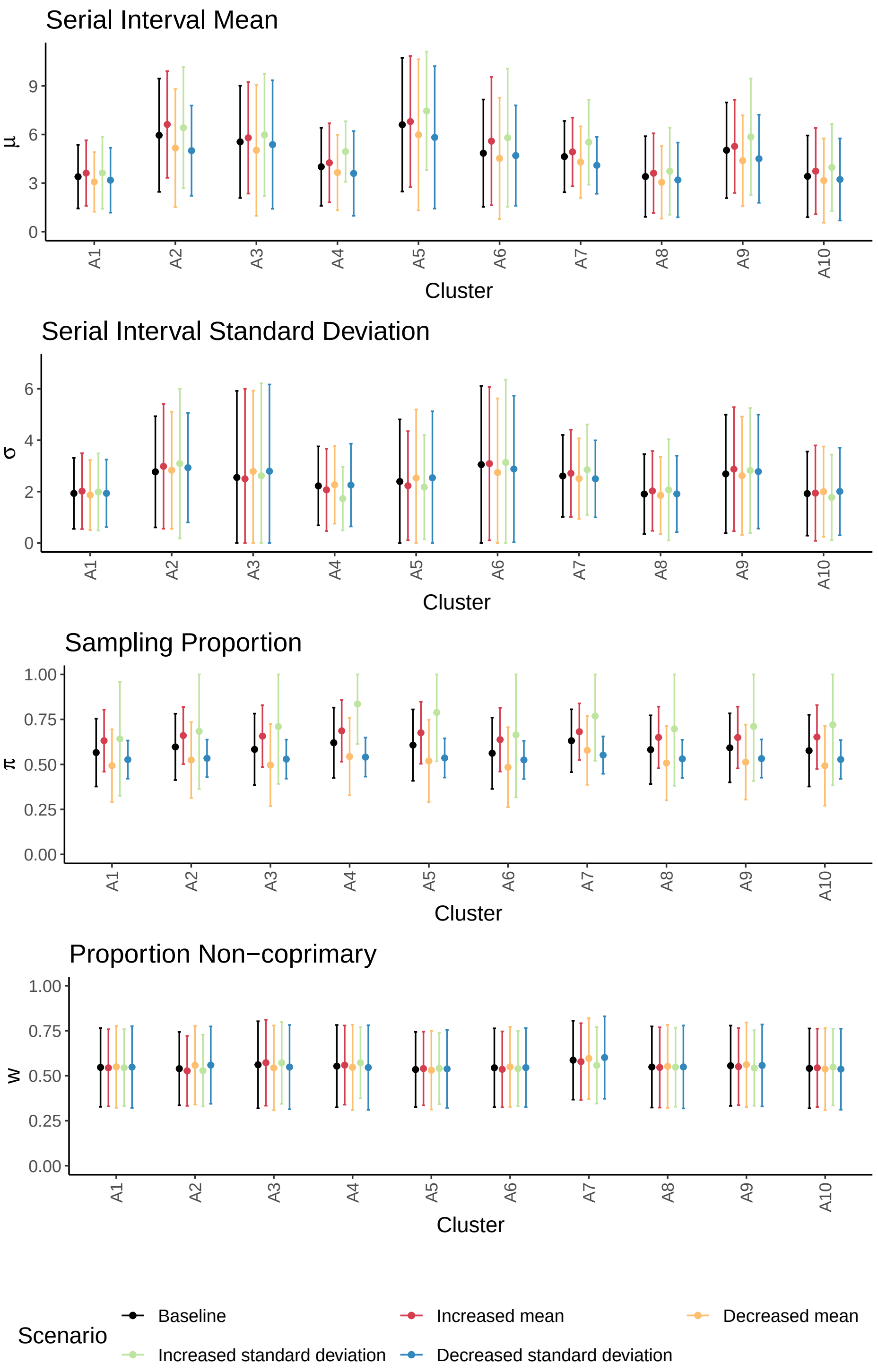

Figure S4: **Results of a sensitivity analysis for the prior distribution of π, for wave 1 clusters with size as defined in Table 1 and 100 sampled transmission networks.** Estimates of model parameters (μ, σ, π, w) under the 4 alternate prior distributions for π presented in Figure S3, compared to baseline Beta(12,11) prior from main analysis. Prior on w is held fixed at the baseline value. Mean estimates shown as points, and 95% confidence intervals as bars.

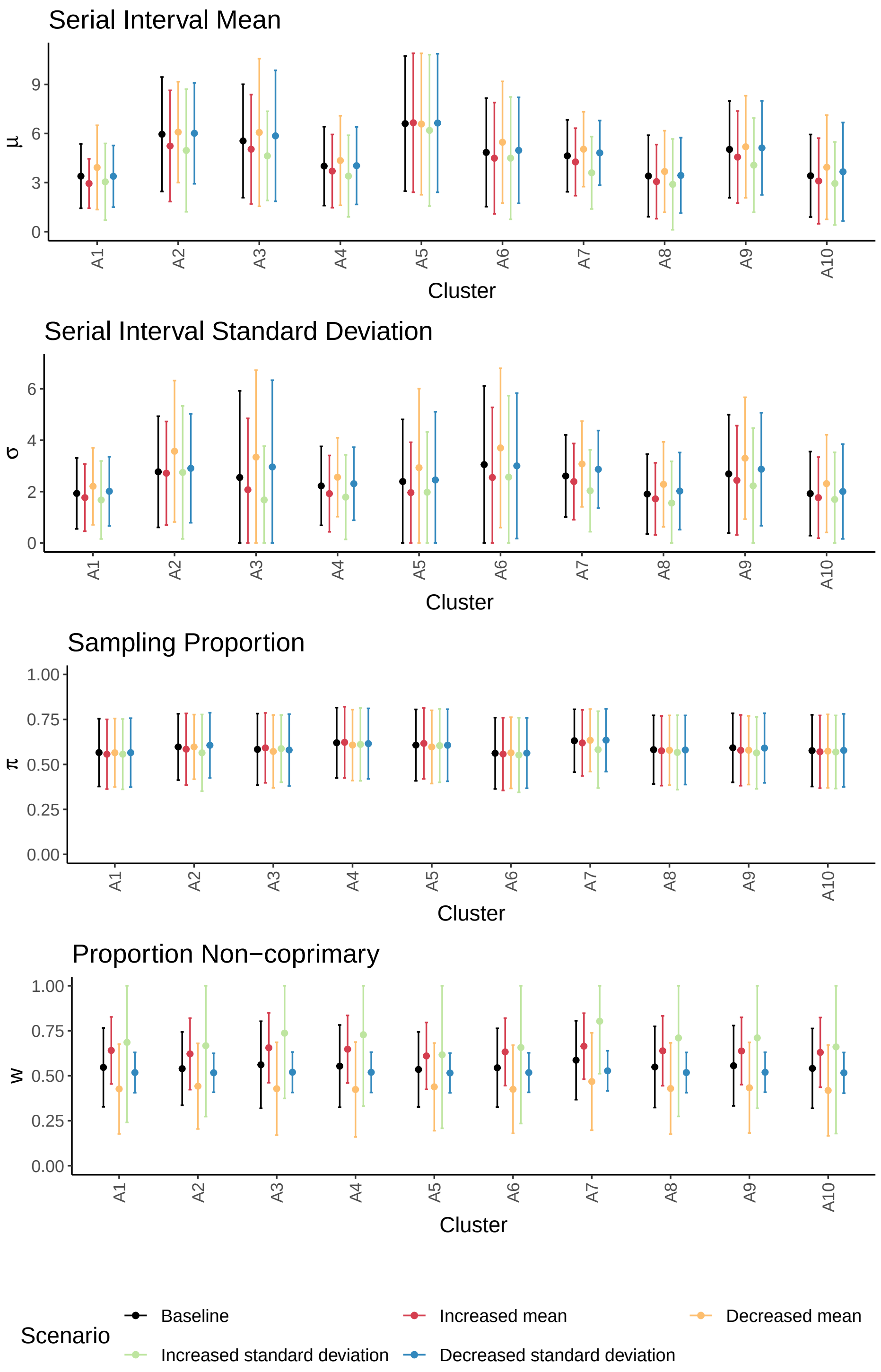

Figure S5: **Results of a sensitivity analysis for the prior distribution of w, for wave 1 clusters with size as defined in Table 1 and 100 sampled transmission networks.** Estimates of model parameters (μ, σ, π, w) under the 4 alternate prior distributions for w presented in Figure S3, compared to baseline Beta(12,11) prior from main analysis. Prior on π is held fixed at the baseline value. Mean estimates shown as points, and 95% confidence intervals as bars.

Table S5: **Seven simulation study scenarios for exploring sensitivity to the prior distribution of π and w.**

Scenario	Beta(a,b)	Mean	SD
Baseline	12, 12	0.5	0.1
Increased mean	18, 12	0.6	0.09
Further increased mean	9, 3	0.75	0.12
Decreased mean	6, 12	0.33	0.11
Further decreased mean	3, 10	0.23	0.11
Increased standard deviation	5, 5	0.5	0.15
Decreased standard deviation	50, 50	0.5	0.05

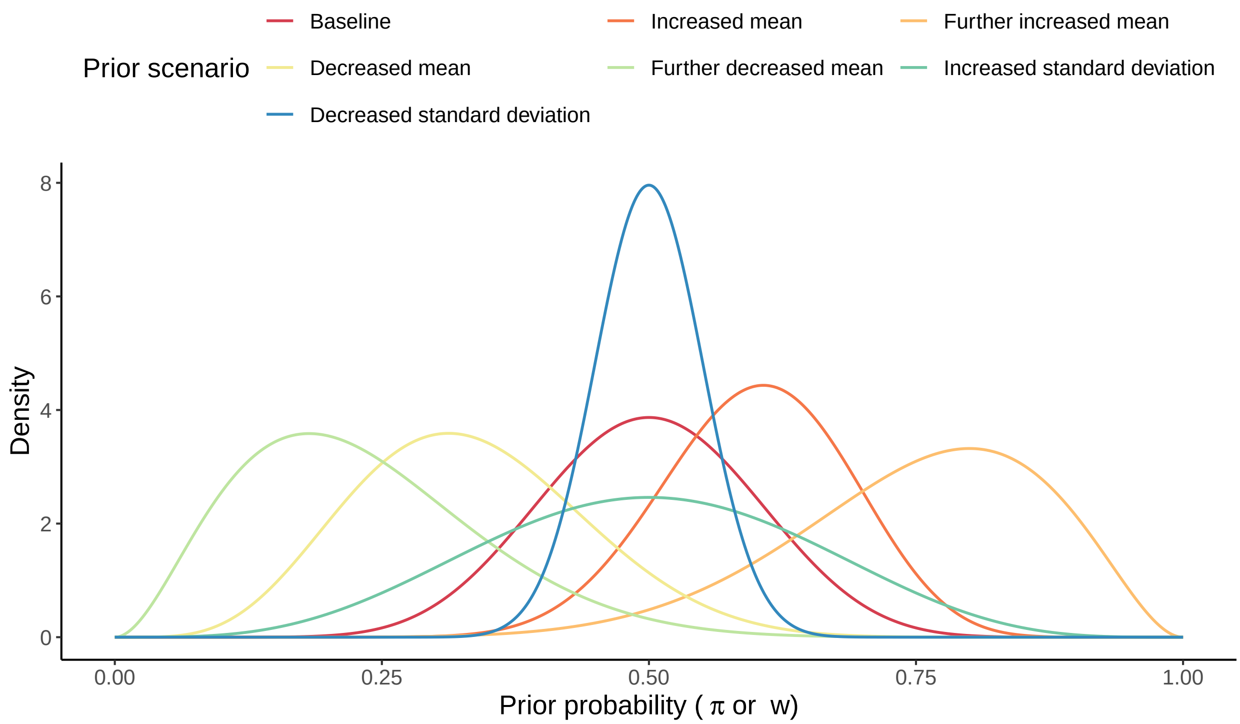

Figure S6: **Prior distributions for parameters π and w used in sensitivity analysis of prior choice.** Scenarios are as defined in Table S5.

The results of this simulation study are presented in Figure S7. We find similar results as in the COVID-19 prior sensitivity analysis: as we vary the priors on π and w, our MAP estimates of these parameters vary somewhat also, but the serial interval mean and standard deviation are relatively robust. Our estimates of the serial interval mean are most affected by a prior underestimation of the sampling rate, suggesting that in future analyses it may be beneficial to select a more optimistic than pessimistic prior. We note that the lack of a 1:1 relationship between π/w and the proportion

of simulated cases that we sample limits the interpretability of this simulation study: we do not know the 'true' values of π and w to compare against, and these will change between different simulated outbreaks and depending upon whom we happen to sample. This may be the cause of the interesting behaviour when we reduce the standard deviation of the priors: we force π and w, perhaps incorrectly, closer to 0.5. In turn, this increases the standard deviation of the direct serial intervals. This would suggest that, even if we are confident in the proportion of cases we sample and sequence, we should not enforce strong priors on π and w because local population dynamics may be highly influential on the transmission network. For example, an unsampled super-spreader causing many coprimary pairs can occur even under strong sampling.

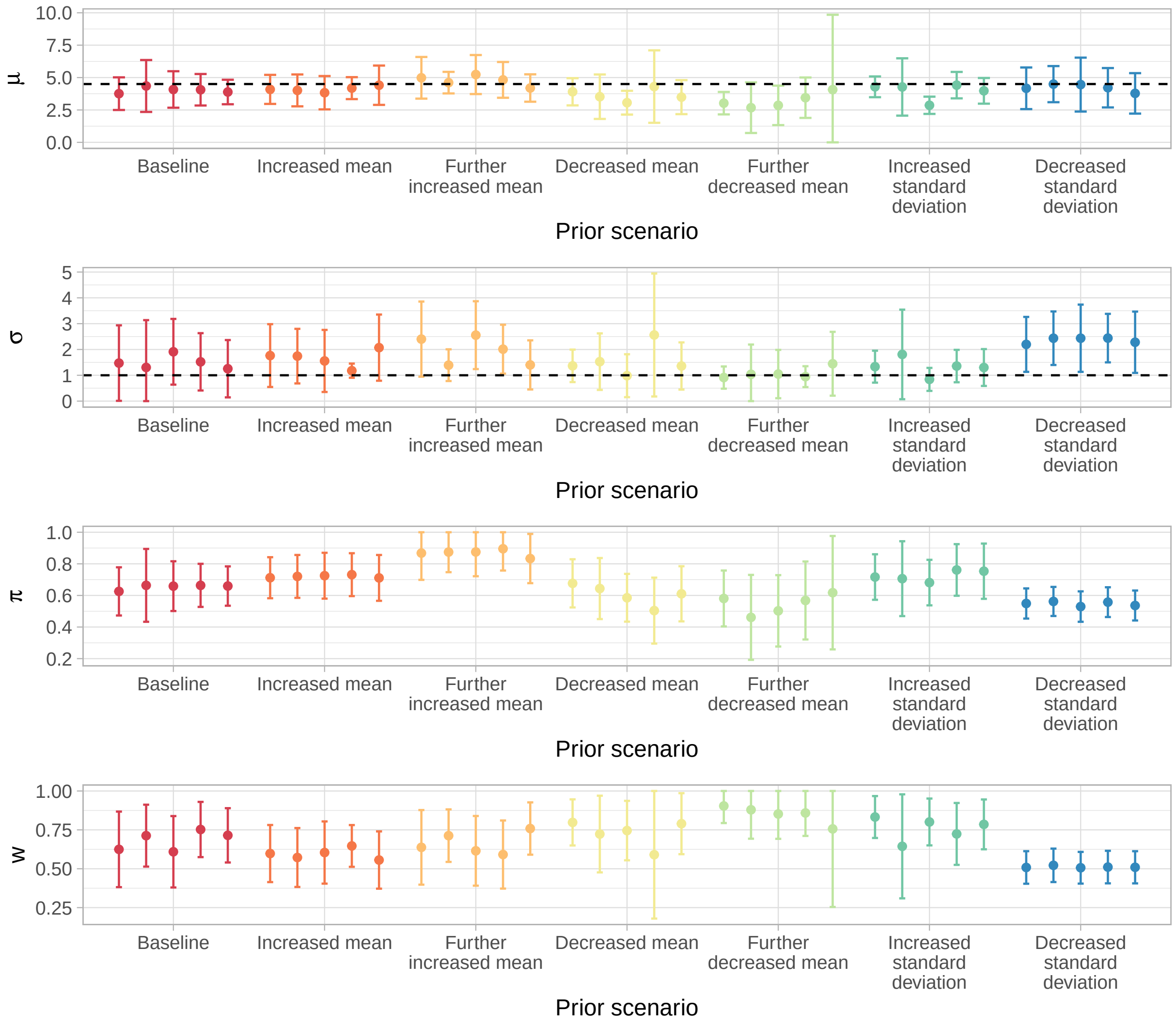

Figure S7: **Results of a sensitivity analysis for the prior distribution of π and w using simulated outbreaks, with population size $n = 200$ and 100 sampled transmission networks.** Estimates of model parameters (μ, σ, π, w) under the 7 prior distributions for π and w presented in Figure S6. Mean estimates shown as points, and 95% confidence intervals as bars. The true serial interval mean and standard deviation are shown as dashed horizontal lines.

S3 Differences in the cluster-specific serial intervals

Although we observed differences in our cluster-specific serial interval estimates in Figure 5, it is not clear if these differences are statistically significant. There are many ways in which distributions can be different (mean, variance, skew): we focus here on the mean of our serial intervals, as this has arguably the largest epidemiological significance and impact to disease control. To investigate this, we test if the 20 cluster-specific mean serial intervals $\hat{\mu}_c$ (from cluster $c = 1, \dots, 20$) are all consistent with being observations of the same (unknown) population-level mean serial interval μ^*. Under H_0, $\mu_1 = \mu_2 = \cdots = \mu_{20} = \mu^*$, where μ_c is the true mean serial interval in cluster c.

Recall that, in our calculation of the confidence intervals in the main text, we assumed that,

$$\hat{\mu}_c \sim \mathcal{N}(\mu_c, \sigma_c),$$

in each cluster c, where σ_c is the standard error of the estimator $\hat{\mu}_c$, calculated through the law of total variance over all sampled transmission networks. Note, this σ_c is not our estimate of the serial interval standard deviation, as in Figure 5. Therefore,

$$\frac{\hat{\mu}_c - \mu_c}{\sigma_c} \sim \mathcal{N}(0, 1).$$

The square of a standard normal distribution is chi-squared, and hence,

$$\frac{(\hat{\mu}_c - \mu_c)^2}{\sigma_c^2} \sim \chi^2(1).$$

Summing over all clusters $c = 1, \dots, m$,

$$\sum_{c=1}^{m} \frac{(\hat{\mu}_c - \mu_c)^2}{\sigma_c^2} \sim \chi^2(m).$$

The true cluster means μ_c are unknown, but recall that under H_0 all μ_c are equal to μ^*. We estimate

the unknown population mean μ^* as the weighted mean of the set of $\hat{\mu}_c$, obtained by maximising $\prod_c \phi(\hat{\mu}_c|\mu^*, \sigma_c)$, where $\phi(x|\mu, \sigma)$ is the Gaussian pdf with mean μ and standard deviation σ. This gives the estimate

$$\hat{\mu^*} = \frac{\sum_c \hat{\mu}_c/\sigma_c^2}{\sum_c 1/\sigma_c^2}.$$

We lose one degree of freedom through this additional constraint on the set of the $\hat{\mu}_c$, and therefore

$$\sum_{c=1}^{m} \frac{(\hat{\mu}_c - \hat{\mu^*})^2}{\sigma_c^2} \sim \chi^2(m-1),$$

forms our test statistic, where $m = 20$ in this case and $\hat{\mu^*} = 4.385$.

For our 20 primary clusters, the value of our test statistic is 19.07, lower than the critical value 30.14 at a 5% level of significance for a $\chi^2(19)$ distribution. We therefore do not have sufficient evidence to reject the null hypothesis, and we cannot conclude that the serial interval distributions in different clusters have different means.

We next look at serial interval means for the wave 2 clusters categorized by exposure site type. As shown in Figure 8, we classify wave 2 cluster sites into either Aged Care, Healthcare, Housing, Packing Plant/Meat processing, or School. (We exclude clusters labelled NA for this analysis.) For each site type we repeat the test we performed above to see if the different cluster types have different mean serial intervals. We find that, with the exception of Aged Care, we can not reject the hypothesis of identical mean serial interval for all clusters within a given site type. Within Aged Care the outlier is B82 which has an unusually low mean estimate with a low standard error estimate. Excluding B82, the clusters in Aged Care appear to have the same mean according to our test. We then estimate for each site type a mean serial interval with a standard error using the method above (excluding B82 for Aged Care). We show the results in Figure S8. Using the same test as for the 20 primary clusters above, we test if the five site types have different serial interval means. We reject the hypothesis of identical serial interval means with $p = 3 \times 10^{-4}$. The biggest outlier is Packing Plant/Meat Processing with a significantly shorter mean serial interval

estimate. Excluding this site type and repeating with the remaining 4, we are unable to reject the hypothesis of identical serial interval means. We conclude that Packing Plant/Meat Processing has significantly lower mean serial interval than other settings, and in particular this is most striking in contrast with Aged Care and Healthcare sites.

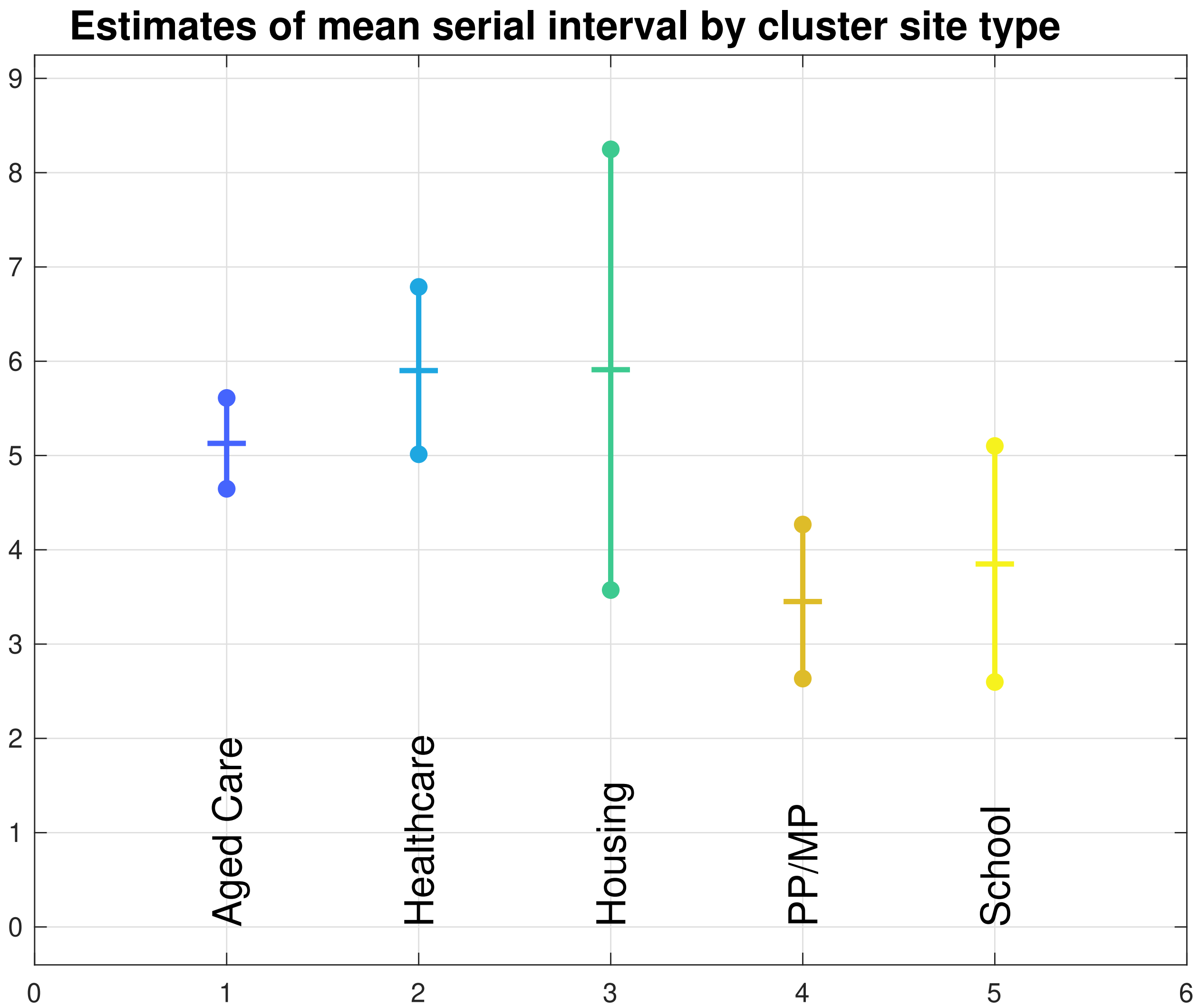

Figure S8: **Estimates of the serial interval mean by cluster site type, for the wave 2 clusters**. All clusters from the same site type are assumed to have the same mean, and cluster B82 was excluded from Aged Care. PP/MP = packing plant/meat processing

We additionally perform a simulation study to explore the ability of our method to distinguish between different length serial intervals. Using the same simulation model as in the main text (see Methods), we simulate 5 outbreaks under each of 5 different mean serial intervals $\mu = 1.2, 2, 3, 4, 5$

days (our simulator requires serial intervals > 1 day). In each scenario, the standard deviation σ is set to 1. We simulate outbreaks in populations of 200 individuals, and down-sample them at random by 50% to approximate a similar setting as our COVID-19 analysis. We accordingly use a Beta(12, 11) prior for π and w with mean 0.52 and standard deviation 0.1. For each simulated outbreak, we sample 100 transmission networks as in the COVID-19 analysis, linearly scaling the genomic distance threshold with the mean serial interval to account for more expected mutations between transmission pairs.

The outbreak scenarios and the model parameter estimates are shown in Figure S9. We find that for $\mu \geq 2$ our method is able to correctly distinguish the serial interval mean. In all cases the true value is within the 95% confidence interval. For $\mu = 1.2$ however, all estimates are biased by the method's assumption of strictly positive serial intervals, artificially reducing the serial interval standard deviation. The true values of π and w would be expected to change between simulated outbreaks, because they are determined by which cases we happen to sample at random. The figure suggests that, as μ increases, more cases are identified as missing intermediates (π) than coprimary infectors ($1-w$). However a test of significant difference in π and w between the different outbreak scenarios, exactly as described for the exposure sites COVID-19 analysis above, does not conclude that they have significantly different estimates of π and w.

On the other hand, we do reject the hypothesis of identical serial interval means in the simulated outbreaks. The outbreaks are grouped into their clusters i.e. different true means, and we repeat the test performed above on the exposure site clusters. The test statistic is 59.4, higher than the critical value 9.49 for a $\chi^2(4)$ distribution at the 5% significance level. There is still evidence of significantly different means when we remove the spurious mean 1.2 days outbreaks from the test, with test statistic 14.95 higher than the $\chi^2(3)$ critical value of 7.815.

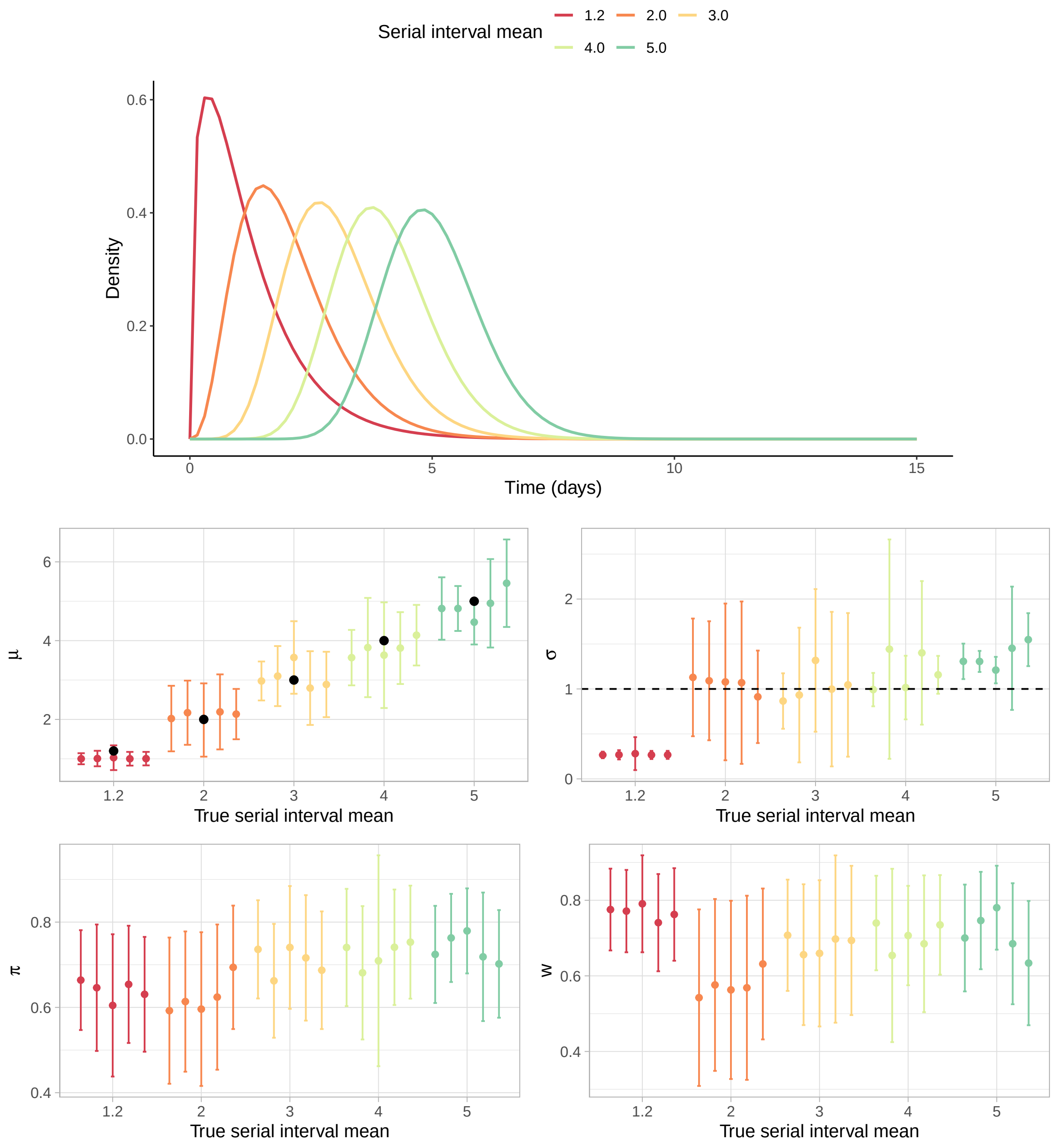

Figure S9: **Simulation study under varying mean serial interval, for simulated outbreaks in a population of size $n = 200$ and 100 sampled transmission networks** Top: true $\Gamma(\mu, 1)$ serial interval distributions simulated from. Bottom: model parameter estimates for 5 simulated outbreaks under each of the 5 scenarios for true mean serial interval μ. Mean estimates shown as points and 95% confidence intervals as bars. True values of μ shown as black points, true value of $\sigma = 1$ in all scenarios shown as black dashed line.

S4 Analysis of prior/likelihood influence

In order to quantify the role of the priors for parameters π and w in our analysis, we calculate the Kullback-Leibler (KL) divergence as a method to informally assess the relative influence of the prior and likelihood on our maximized posterior distributions. The KL divergence $D_{\mathrm{KL}}(P, Q)$ measures the (non-symmetric) divergence, or entropy, between two distributions P and Q. It can be seen as a measure of the information gained by using the posterior distribution P in place of our original prior beliefs Q (i.e. the likelihood information $D_{\mathrm{KL}}(P, Q)$), or conversely the information gained by maximising the posterior instead of simply the likelihood (i.e. the prior information $D_{\mathrm{KL}}(P, L)$) [13].

We calculate the likelihood information for π, $D_{\mathrm{KL}}(P_\pi, Q_\pi)$, and the prior information for π, $D_{\mathrm{KL}}(P_\pi, L_\pi)$, for each of 10 randomly selected transmission networks from each of the 10 wave 1 clusters, where P_π is the marginal posterior for π, L_π is the normalized marginal likelihood for π and Q_π is π's prior distribution. We repeat for w. By comparing $D_{\mathrm{KL}}(P_\pi, Q_\pi)$ to $D_{\mathrm{KL}}(P_\pi, L_\pi)$, and $D_{\mathrm{KL}}(P_w, Q_w)$ to $D_{\mathrm{KL}}(P_w, L_w)$ (holding all other parameters at their MAP values to get the marginals), we assess the relative influence of the likelihood and prior, respectively, on the posterior distribution. We define the relative influence of the prior for π, for example, as

$$D_{\mathrm{KL}}(P_\pi, L_\pi)/(D_{\mathrm{KL}}(P_\pi, L_\pi) + D_{\mathrm{KL}}(P_\pi, Q_\pi)),$$

and correspondingly for the likelihood.

The results of this experiment are shown in Figure S10, averaged over the 10 sampled networks per cluster. As expected from the main text analysis, we find that the priors on π and w are strongly influential on the posterior distribution, contributing around 75% of our relative influence score. Several clusters have a larger relative influence from the likelihood. Primarily, A7 (likelihood relative influence 54% for π and 34% for w), as can be visually identified pulling away from the

prior in Figure 5, and A2 (likelihood relative influence 52% for π and 34% for w), where the likelihood and prior are in relative agreement. These are the two largest wave 1 clusters (Table 1), and so it follows logically that the data would be more informative, but also suggests that with increased surveillance and therefore larger clusters, the reliance on prior assumptions could be minimized. As we do not assume any prior distributions for serial interval parameters μ and σ, the relative influence of their marginal likelihoods would be 1.0.

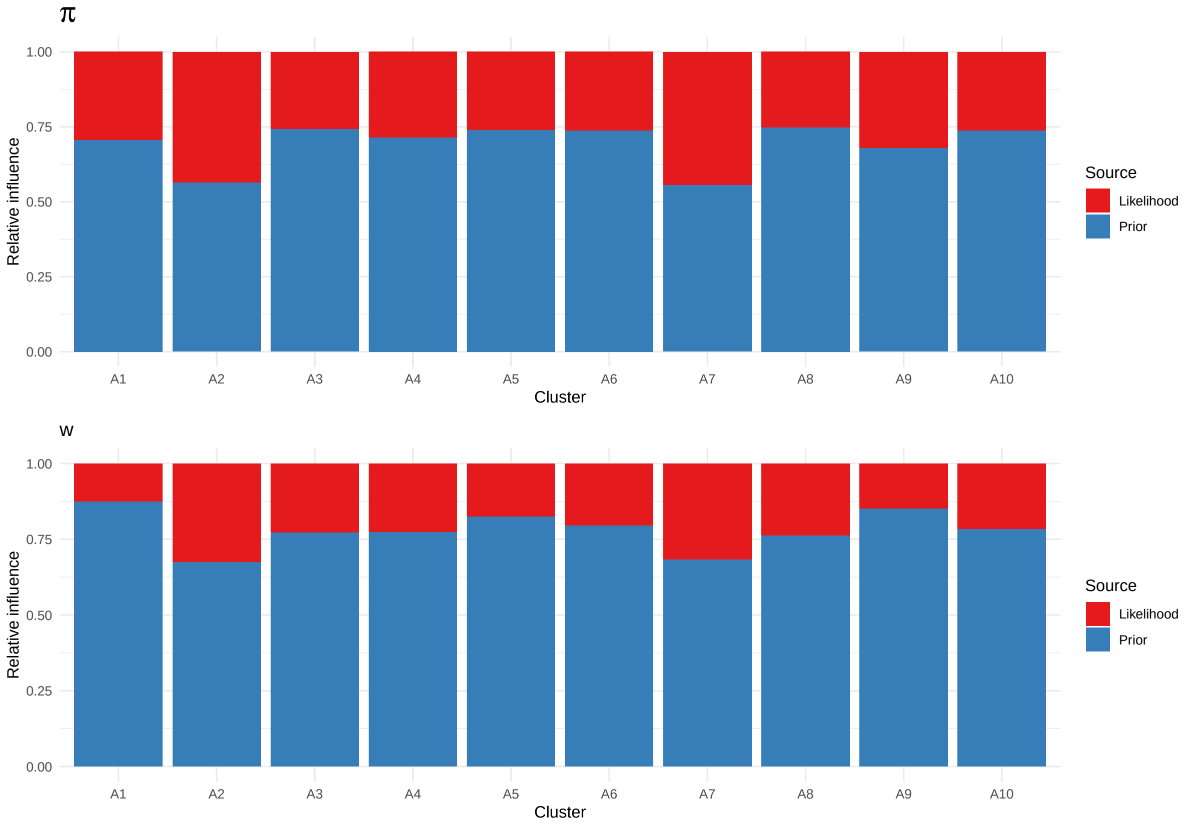

Figure S10: **Kullback-Leibler (KL) divergence compares the relative influence of the prior and likelihood distributions when estimating parameters π and w.** Presented results per wave 1 cluster are averaged over 10 plausible transmission networks randomly selected from the set of networks sampled in the main analysis.

S5 Comparison to contact-defined clusters

To further validate our genomic approach, we apply the same estimation procedure to contact-defined clusters and transmission networks. These contact-based clusters are defined as the connected components of a network created from contact tracing data, where an edge in the contact network indicates either a known direct contact or a shared exposure site. We again exclude any clusters with fewer than 15 cases. We focus on only the wave 1 data for this analysis, as the contact tracing data showed higher resolution with less uncertainty. The resulting clusters are shown in Figure S11.

We then define a transmission cloud as the set of all plausible transmission pairs, again using contact data in place of viral sequences. Here, a plausible transmission pair is two cases such that the time between symptom onset dates of the putative infector and infectee pair is positive and less than 35 days (as before, but with no genomic element). As before, this means that each infectee may have more than one plausible infector. Transmission networks are sampled from the transmission cloud by preferentially sampling from edges which represent confirmed contacts (as opposed to shared exposure locations). We sample 100 transmission networks per cluster. We estimate the serial interval in each cluster, using a beta-distributed prior for π and w. We use a $\text{Beta}(9, 2.5)$ prior distribution (mean 0.78) to reflect that a larger proportion of Victorian COVID-19 cases were contact traced than genomically sequenced.

The cluster-specific estimates for the serial interval parameters are shown in Figures S12, with the resulting distributions shown in Figure S13. Figure S14 shows the comparison of these results with the published estimates in Table S2. For those contact clusters sharing at least 50% of cases with a genomically-defined cluster (as shown in Figure S11), we compare the resulting estimates in Figure 9 in the main text.

In Figure S15, we compare the probabilities with which each plausible infector is chosen to be the ancestor of each infectee in the genomic analysis (as defined in Methods), against the contact

tracing data, where available. Each infectee with at least one direct traced contact is shown as a panel, and all of their plausible infectors are shown with their assigned probability from the genomic analysis (y-axis) and contact type from the contact tracing data (colour). For most infectees, there are many possible infectors, the majority of whom do not have any contact-tracing links. However, note that lack of an identified contact link does not necessarily correspond to lack of a link in reality. In many cases, we see that infectors with known contact to the infectee (shown in red) are assigned a higher probability, relative to the other potential infectors. The same is true for shared exposure sites to some extent, though less reliably. For example, out of 51 infectors with identified contact links to their plausible infectee, 40 are among the highest ranked infectors. For infector-infectee pairs with shared exposure site, 70/133 plausible infectors are among the highest ranked.

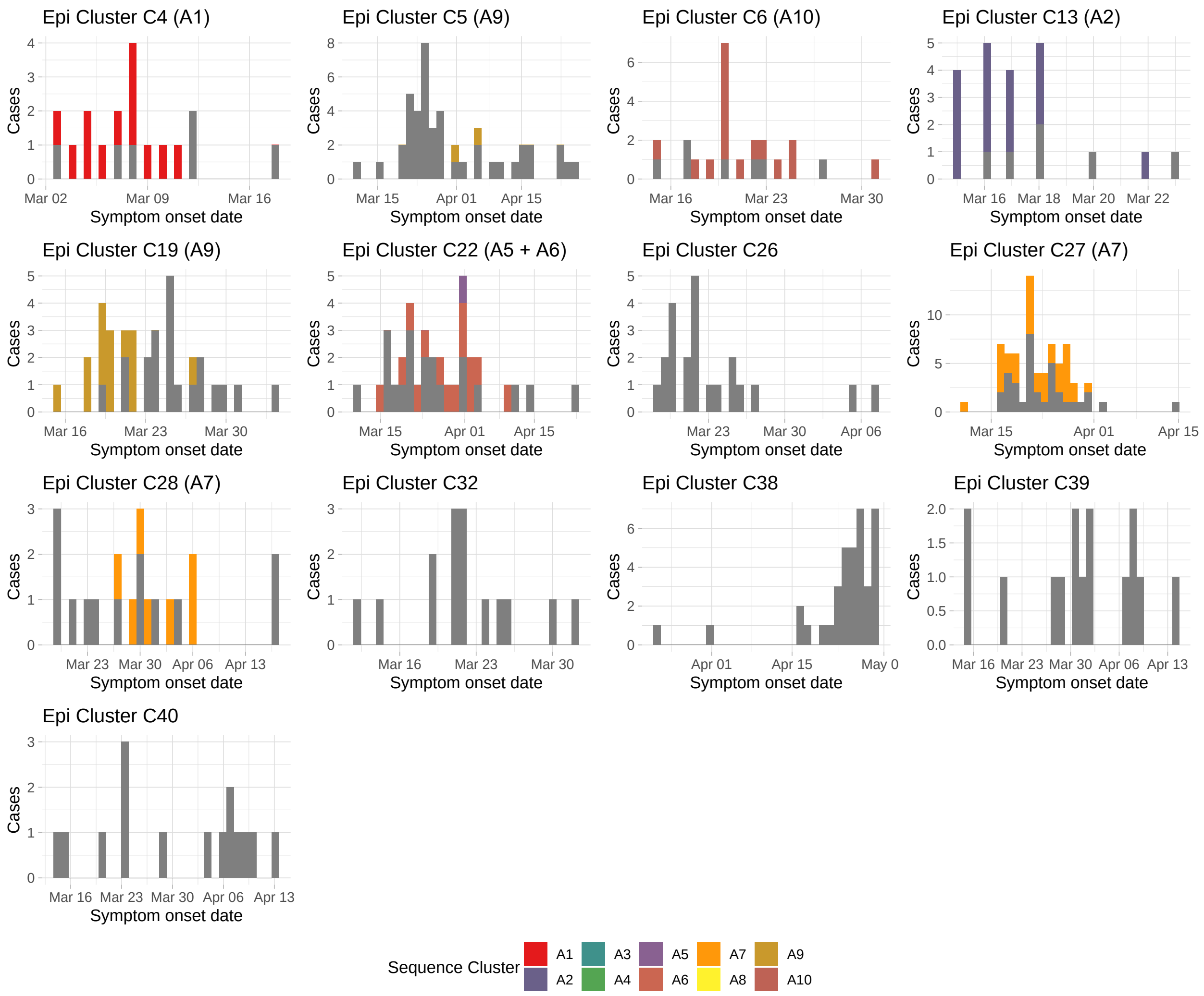

Figure S11: **Summary of contact-defined clusters for wave 1 data**. Cases plotted by reported symptom onset date, for all identified contact clusters with at least 15 cases. Cases are coloured by their cluster membership in the genomically defined clusters A1–A10 from the main text, with grey indicating a case was not included in any of A1–A10. Brackets in plot titles also indicate which contact clusters have cases from which genomic clusters.

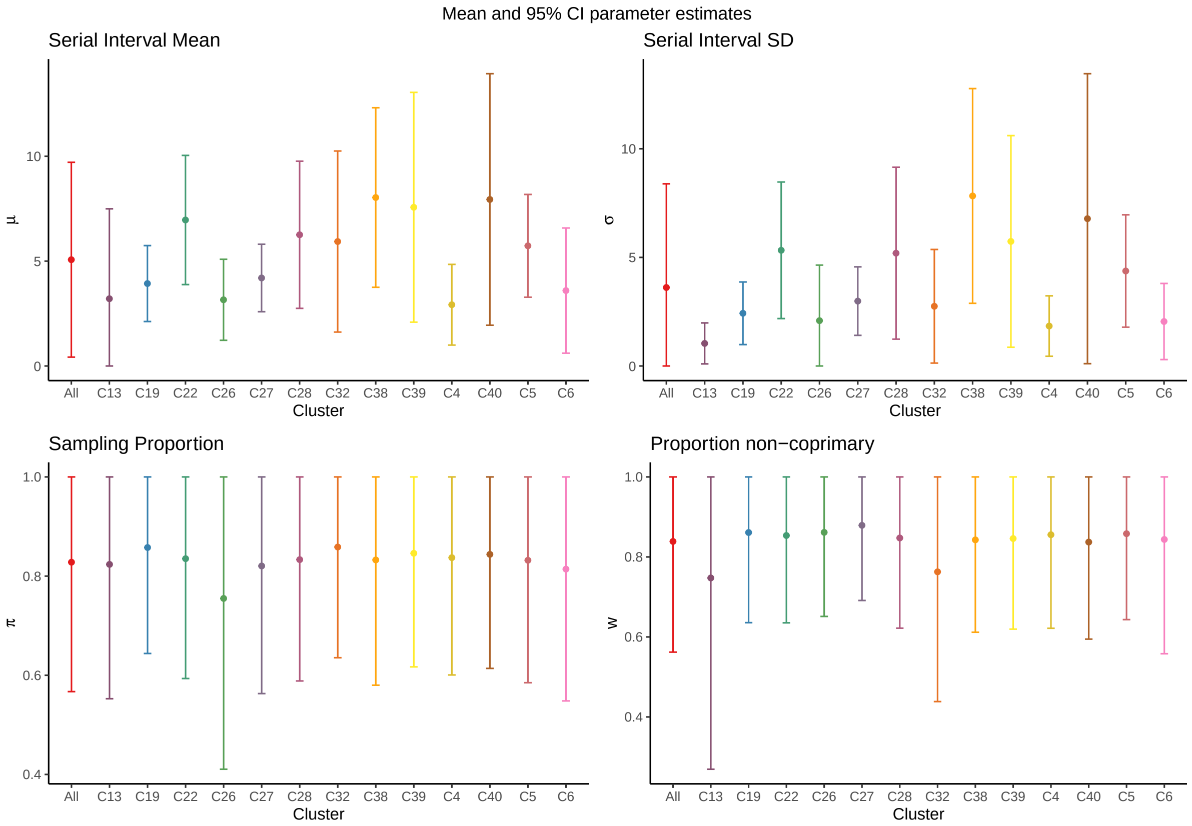

Figure S12: **Estimates of model parameters per contact-defined cluster, with cluster sizes as shown in Figure S11 and 100 sampled transmission networks.** Mean estimates shown as points and 95% confidence intervals as bars.

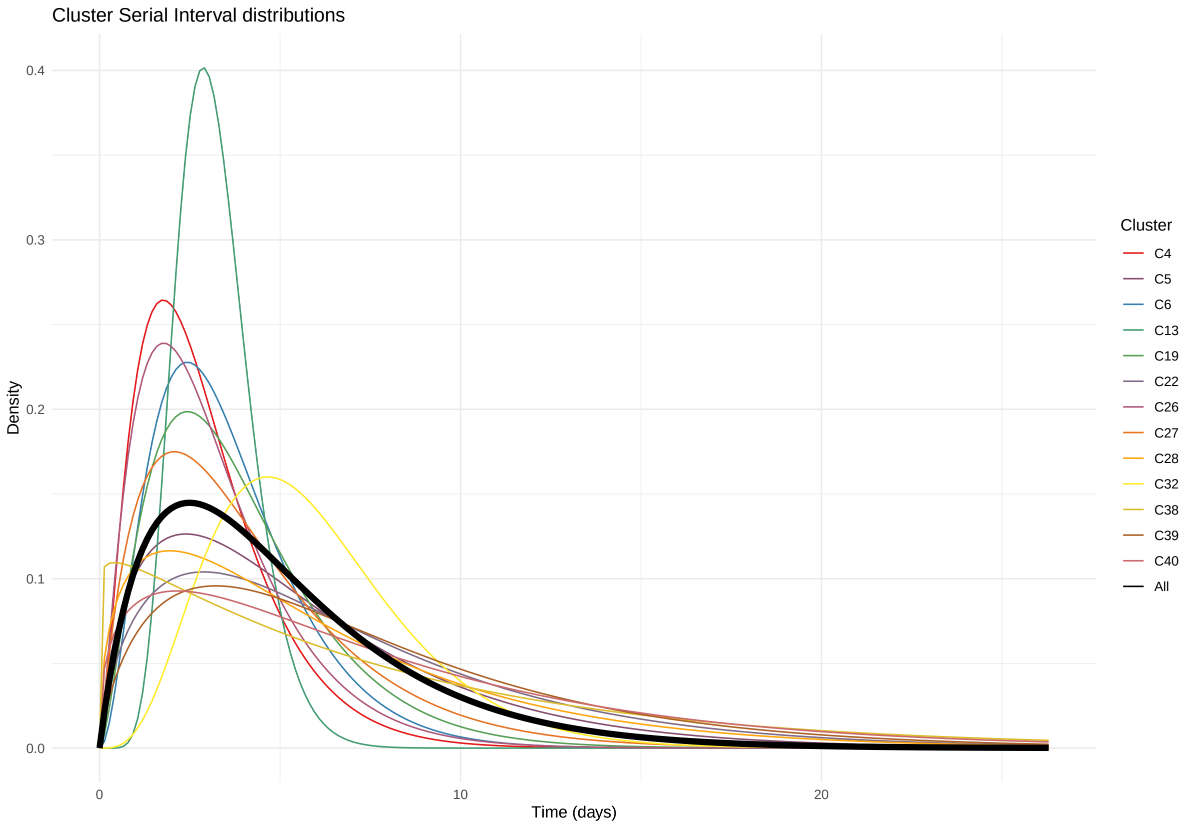

Figure S13: **Estimate of serial interval distribution per contact-defined cluster.** Black (bold) curve indicates the pooled estimate across all clusters in the analysis.

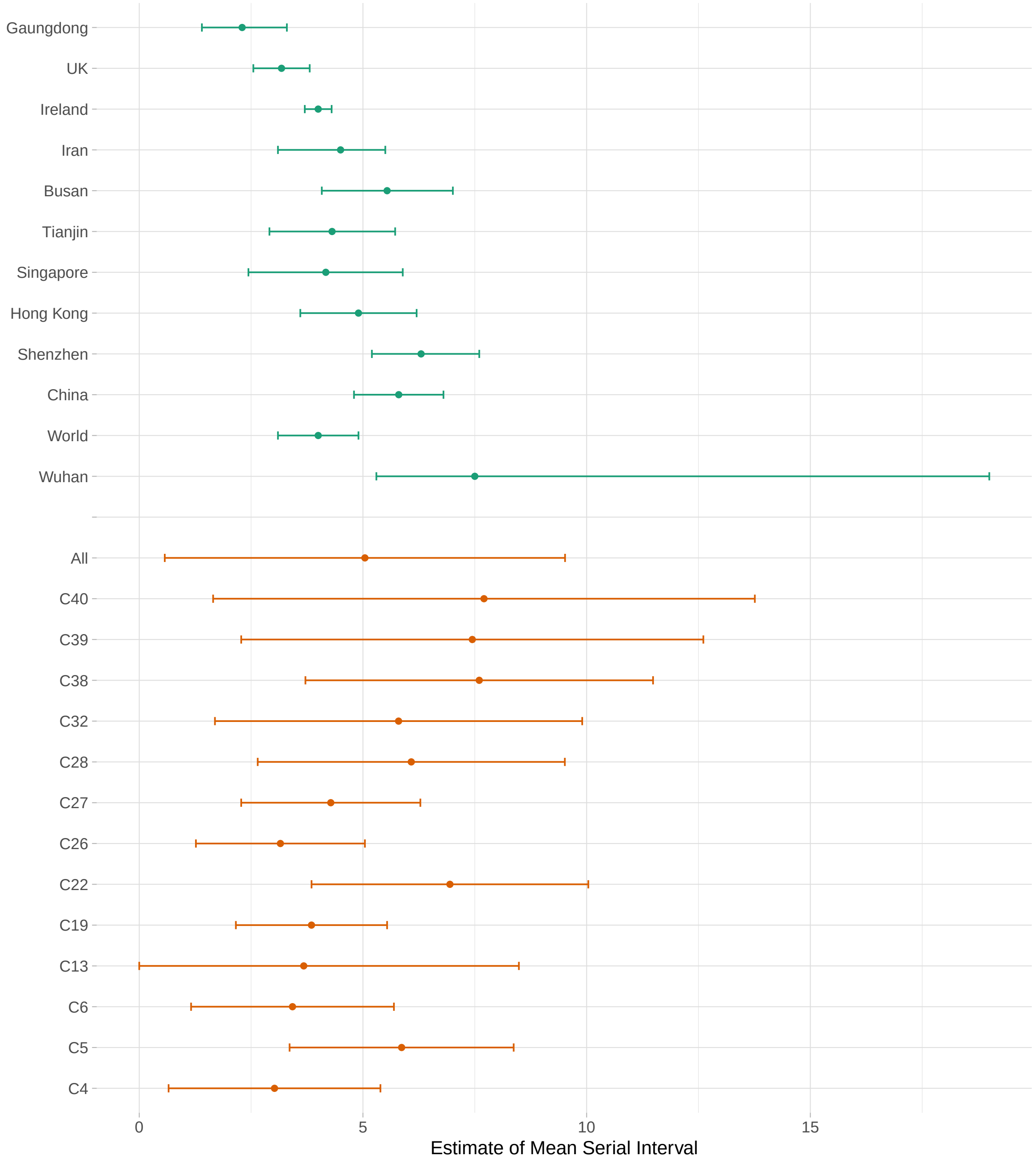

Figure S14: **Comparison of the mean serial interval for the contact-defined clusters shown in Figure S11, with the published results listed in Table S2**. Each point indicates the estimate for the mean serial interval, with bars indicating the 95% confidence intervals. The first 12 rows (green) show the previously published estimates, while the remaining 14 rows (orange) show the results from this analysis. Contact clusters have cluster sizes as shown in Figure S11, and 100 sampled transmission networks. Sample sizes of previously published outbreaks are shown in Table S2.

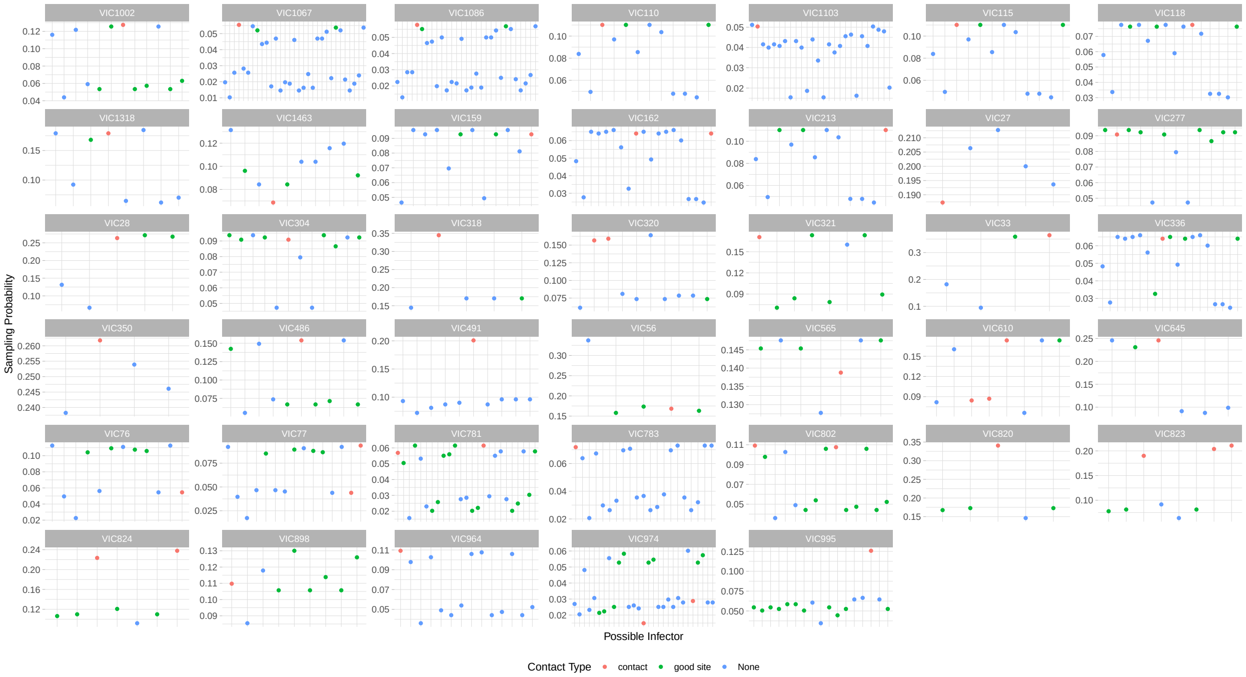

Figure S15: **Comparison of the inferred infector-infectee pair probabilities from the genomic analysis, against known contact links.** Each panel represents an infectee for which at least one direct contact is known. The sampling probabilities for each plausible infector of that case are calculated as described in Methods. Each possible infector-infectee pair is coloured according to the available contact data: red indicates a direct contact match, green indicates a shared exposure site only, and blue indicates that there was no known link between the pair in the contact tracing data.

References

[1] Torsten Seemann, Courtney R Lane, Norelle L Sherry, Sebastian Duchene, Anders Gonçalves da Silva, Leon Caly, Michelle Sait, Susan A Ballard, Kristy Horan, Mark B Schultz, et al. Tracking the COVID-19 pandemic in Australia using genomics. *Nature communications*, 11(1):1–9, 2020.

[2] Qun Li, Xuhua Guan, Peng Wu, Xiaoye Wang, Lei Zhou, Yeqing Tong, Ruiqi Ren, Kathy SM Leung, Eric HY Lau, Jessica Y Wong, et al. Early transmission dynamics in Wuhan, China, of novel coronavirus–infected pneumonia. *New England journal of medicine*, 2020.

[3] Hiroshi Nishiura, Natalie M Linton, and Andrei R Akhmetzhanov. Serial interval of novel coronavirus (COVID-19) infections. *International journal of infectious diseases*, 93:284–286, 2020.

[4] Xi He, Eric HY Lau, Peng Wu, Xilong Deng, Jian Wang, Xinxin Hao, Yiu Chung Lau, Jessica Y Wong, Yujuan Guan, Xinghua Tan, et al. Temporal dynamics in viral shedding and transmissibility of COVID-19. *Nature medicine*, 26(5):672–675, 2020.

[5] Qifang Bi, Yongsheng Wu, Shujiang Mei, Chenfei Ye, Xuan Zou, Zhen Zhang, Xiaojian Liu, Lan Wei, Shaun A Truelove, Tong Zhang, et al. Epidemiology and transmission of COVID-19 in 391 cases and 1286 of their close contacts in Shenzhen, China: a retrospective cohort study. *The Lancet Infectious Diseases*, 20(8):911–919, 2020.

[6] Shi Zhao, Daozhou Gao, Zian Zhuang, Marc KC Chong, Yongli Cai, Jinjun Ran, Peihua Cao, Kai Wang, Yijun Lou, Weiming Wang, et al. Estimating the serial interval of the novel coronavirus disease (COVID-19): a statistical analysis using the public data in Hong Kong from January 16 to February 15, 2020. *Frontiers in Physics*, 8:347, 2020.

[7] Lauren C Tindale, Jessica E Stockdale, Michelle Coombe, Emma S Garlock, Wing Yin Venus Lau, Manu Saraswat, Louxin Zhang, Dongxuan Chen, Jacco Wallinga, and Caroline Colijn. Evidence for transmission of COVID-19 prior to symptom onset. *Elife*, 9:e57149, 2020.

[8] Hyunjin Son, Hyojung Lee, Miyoung Lee, Youngduck Eun, Kyounghee Park, Seungjin Kim, Wonseo Park, Sora Kwon, Byoungseon Ahn, Dongkeun Kim, et al. Epidemiological characteristics of and containment measures for COVID-19 in Busan, Korea. *Epidemiology and health*, 42, 2020.

[9] Fariba Zare, Mohammad Hassan Emamian, Reza Chaman, Marzieh Rohani-Rasaf, Shahrbanoo Goli, Solmaz Talebi, Ali Hosseinzadeh, and Ahmad Khosravi. Serial interval distribution of COVID-19 among Iranian reported confirmed cases. *International Journal of Health Studies*, 7(4), 2021.

[10] Conor G McAloon, Patrick Wall, John Griffin, Miriam Casey, Ann Barber, Mary Codd, Eamonn Gormley, Francis Butler, Locksley L McV Messam, Cathal Walsh, et al. Estimation of the serial interval and proportion of pre-symptomatic transmission events of COVID- 19 in Ireland using contact tracing data. *BMC public health*, 21(1):1–9, 2021.

[11] Cyril Roman Geismar, Ellen Fragaszy, Vincent Grigori Nguyen, Wing Lam Erica Fong, Madhumita Shrotri, Sarah Beale, Alison Rodger, Vasileios Lampos, Thomas Edward Byrne, Jana Kovar, et al. Serial interval of COVID-19 and the effect of Variant B. 1.1. 7: analyses from a prospective community cohort study (Virus Watch). *medRxiv*, 2021.

[12] Meng Zhang, Jianpeng Xiao, Aiping Deng, Yingtao Zhang, Yali Zhuang, Ting Hu, Jiansen Li, Hongwei Tu, Bosheng Li, Yan Zhou, et al. Transmission Dynamics of an Outbreak of the COVID-19 Delta Variant B.1.617.2 — Guangdong Province, China, May–June 2021. *China CDC Weekly*, 3(27):584–586, 2021.

[13] Giri Gopalan. Quantification of observed prior and likelihood information in parametric bayesian modeling. *arXiv preprint arXiv:1511.01214*, 2015.
