# Supplementary material for: Genomic epidemiology offers high resolution estimates of serial intervals for COVID-19
Source: Nat Commun. 2023 Aug 10;14:4830. doi: 10.1038/s41467-023-40544-y (PMC10415581; doi:10.1038/s41467-023-40544-y)
Supplement: Supplementary file 3 — Description of Additional Supplementary Files [file 41467_2023_40544_MOESM3_ESM.pdf]

Description of Additional Supplementary Files

File Name: Supplementary Data 1
Description: Acknowledgments to the submitting laboratories for the GISAID sequences
