# Supplementary material for: Genomic epidemiology offers high resolution estimates of serial intervals for COVID-19
Source: Nat Commun. 2023 Aug 10;14:4830. doi: 10.1038/s41467-023-40544-y (PMC10415581; doi:10.1038/s41467-023-40544-y)
Supplement: Supplementary file 5 — Reporting Summary [file 41467_2023_40544_MOESM5_ESM.pdf]

nature portfolio

Corresponding author(s): Jessica E Stockdale

Last updated by author(s): Jun 30, 2023

Reporting Summary

Nature Portfolio wishes to improve the reproducibility of the work that we publish. This form provides structure for consistency and transparency in reporting. For further information on Nature Portfolio policies, see our Editorial Policies and the Editorial Policy Checklist.

Statistics

For all statistical analyses, confirm that the following items are present in the figure legend, table legend, main text, or Methods section.

n/a	Confirmed	
☐	☒	The exact sample size (n) for each experimental group/condition, given as a discrete number and unit of measurement
☒	☐	A statement on whether measurements were taken from distinct samples or whether the same sample was measured repeatedly
☐	☒	The statistical test(s) used AND whether they are one- or two-sided<br>*Only common tests should be described solely by name; describe more complex techniques in the Methods section.*
☐	☒	A description of all covariates tested
☐	☒	A description of any assumptions or corrections, such as tests of normality and adjustment for multiple comparisons
☐	☒	A full description of the statistical parameters including central tendency (e.g. means) or other basic estimates (e.g. regression coefficient) AND variation (e.g. standard deviation) or associated estimates of uncertainty (e.g. confidence intervals)
☐	☒	For null hypothesis testing, the test statistic (e.g. F, t, r) with confidence intervals, effect sizes, degrees of freedom and P value noted<br>*Give P values as exact values whenever suitable.*
☐	☒	For Bayesian analysis, information on the choice of priors and Markov chain Monte Carlo settings
☒	☐	For hierarchical and complex designs, identification of the appropriate level for tests and full reporting of outcomes
☒	☐	Estimates of effect sizes (e.g. Cohen's d, Pearson's r), indicating how they were calculated

Our web collection on statistics for biologists contains articles on many of the points above.

Software and code

Policy information about availability of computer code

Data collection	No software was used. Data collected as part of routine public health operations (details in Methods)
Data analysis	Clustering: IQtree v1.6.12 and ClusterPicker v1.2.3 tools<br>Phylogeny building (figure 2): github.com/MDU-PHL/kovid-trees-nf using GOALIGN v.0.3.4, RAxML-NG v.1.0.2, FastTree v.2.1.10, GOTREE v.0.4.1, clipkit v.1.1.3<br>All other analysis: R version 4.1.0 including packages outbreaker v1.1.8, ape v5.5. Repository github.com/jessicastockdale/genomicSls contains code to reproduce all remaining figures and results in main text using genomic data.

For manuscripts utilizing custom algorithms or software that are central to the research but not yet described in published literature, software must be made available to editors and reviewers. We strongly encourage code deposition in a community repository (e.g. GitHub). See the Nature Portfolio guidelines for submitting code & software for further information.

Data

Policy information about availability of data

All manuscripts must include a data availability statement. This statement should provide the following information, where applicable:

- Accession codes, unique identifiers, or web links for publicly available datasets
- A description of any restrictions on data availability
- For clinical datasets or third party data, please ensure that the statement adheres to our policy

The data, comprised of GISAID accession numbers, originating/submitting laboratories, symptom onset dates and cluster identifiers for all samples used in this study, are available at github.com/jessicastockdale/genomicSIs [20]. Acknowledgments to the submitting laboratories for the GISAID sequences are available as supplementary data. Wuhan reference genome, Genbank MN908947.3 [https://www.ncbi.nlm.nih.gov/nuccore/MN908947], was used in sequence mapping. Contact tracing data used in supplementary analysis is protected and not publicly available for participant privacy.

Research involving human participants, their data, or biological material

Policy information about studies with human participants or human data. See also policy information about sex, gender (identity/presentation), and sexual orientation and race, ethnicity and racism.

Reporting on sex and gender	Neither participant sex nor gender were provided as part of this study. Data represent all detected and genomically sequenced COVID-19 cases in the region, with no sampling or further recruitment. Study and results are not specific to any particular sex or gender
Reporting on race, ethnicity, or other socially relevant groupings	This study does not include reporting on race or ethnicity. Exposure sites and confirmed contacts were used in epidemiological analyses, these are fully anonymized self-reported physical sites attended by participants and contacts between participants, respectively.
Population characteristics	Data represent all detected and genomically sequenced COVID-19 cases in Victoria, Australia during the periods 6th Jan - 14th Apr 2020 and 1st Jun - 28th Oct 2020, collected as part of routine public health operations. For this study, only anonymized genomic sequence, sequence sampling date, symptom onset date and exposure sites/contact information (wave 2 only) were provided by public health, for privacy reasons no further participant characteristics are made available.
Recruitment	Participants were not recruited, instead all data was collected as part of routine public health operations in testing and detecting cases of COVID-19. We note that individuals who are less likely to be identified as part of these operations may be under-represented in the data e.g. those less likely to present for testing or be contacted during contact tracing.
Ethics oversight	Data were collected in accordance with the Victorian Public Health and Wellbeing Act 2008. Ethical approval was received from the University of Melbourne Human Research Ethics Committee (study number 1954615.3)

Note that full information on the approval of the study protocol must also be provided in the manuscript.

Field-specific reporting

Please select the one below that is the best fit for your research. If you are not sure, read the appropriate sections before making your selection.

[x] Life sciences [ ] Behavioural & social sciences [ ] Ecological, evolutionary & environmental sciences

For a reference copy of the document with all sections, see nature.com/documents/nr-reporting-summary-flat.pdf

Life sciences study design

All studies must disclose on these points even when the disclosure is negative.

Sample size	We selected all samples collected by Victorian public health in the time frame of the study that were sequenced with sufficient quality (see below). No sample size calculation was performed: to present an accurate representation of the population, all available samples were sequenced and none with sufficient quality were excluded
Data exclusions	Pre-established exclusion criteria were as follows: <95% genome recovered, >25 SNPs from the Wuhan-Hu-1 reference genome, >300 ambiguous bases, duplicated sample, missing symptom onset date or no recorded exposure site (wave 2 only).
Replication	Not relevant, no designed experiment performed
Randomization	Not relevant. All samples were subject to the same procedures and analysis
Blinding	Not relevant, study involved no group allocation. Authors were blinded from all patient and epidemiological data other than that described in the manuscript

Reporting for specific materials, systems and methods

We require information from authors about some types of materials, experimental systems and methods used in many studies. Here, indicate whether each material, system or method listed is relevant to your study. If you are not sure if a list item applies to your research, read the appropriate section before selecting a response.

Materials & experimental systems

n/a	Involved in the study
☒	☐ Antibodies
☒	☐ Eukaryotic cell lines
☒	☐ Palaeontology and archaeology
☒	☐ Animals and other organisms
☒	☐ Clinical data
☒	☐ Dual use research of concern
☒	☐ Plants

Methods

n/a	Involved in the study
☒	☐ ChIP-seq
☒	☐ Flow cytometry
☒	☐ MRI-based neuroimaging
